# Acute Pulmonary Edema in Pregnancy – Fluid Overload or Atypical Pre-eclampsia

**DOI:** 10.7759/cureus.19305

**Published:** 2021-11-06

**Authors:** Harpreet Kaur, Madhu Kolli

**Affiliations:** 1 Obstetrics and Gynaecology, Grange University Hospital, Cwmbran, GBR

**Keywords:** pulmonary edema, chronic constipation, bowel washout, fluid overload, pre eclampsia

## Abstract

Acute pulmonary edema in pregnancy is a rare but life-threatening condition with high maternal and perinatal morbidity and mortality. Here we discuss a case of acute pulmonary edema in an antenatal woman with pregnancy complicated by chronic severe constipation, highlighting the importance of the need for close liaison between obstetricians and other specialties in the management of pregnant women.

## Introduction

Acute pulmonary edema in pregnancy is a rare but life-threatening condition with high maternal and perinatal morbidity and mortality. Estimated rates of acute pulmonary edema in pregnancy are variable, ranging from as low as 0.08% to as high as 1.5% [1]. The wide ranges reported are due to the poor reporting of maternal morbidity and lack of minimal reporting datasets of key outcomes in pregnancy and the postpartum period [2]. The most common cause of acute pulmonary edema in pregnancy is in association with severe pre-eclampsia. Other causes include peripartum cardiomyopathy, multiple pregnancy, infections, and fluid overload. Here we discuss a case of acute pulmonary edema in an antenatal woman with pregnancy complicated by chronic severe constipation.

## Case presentation

**Figure 1:**
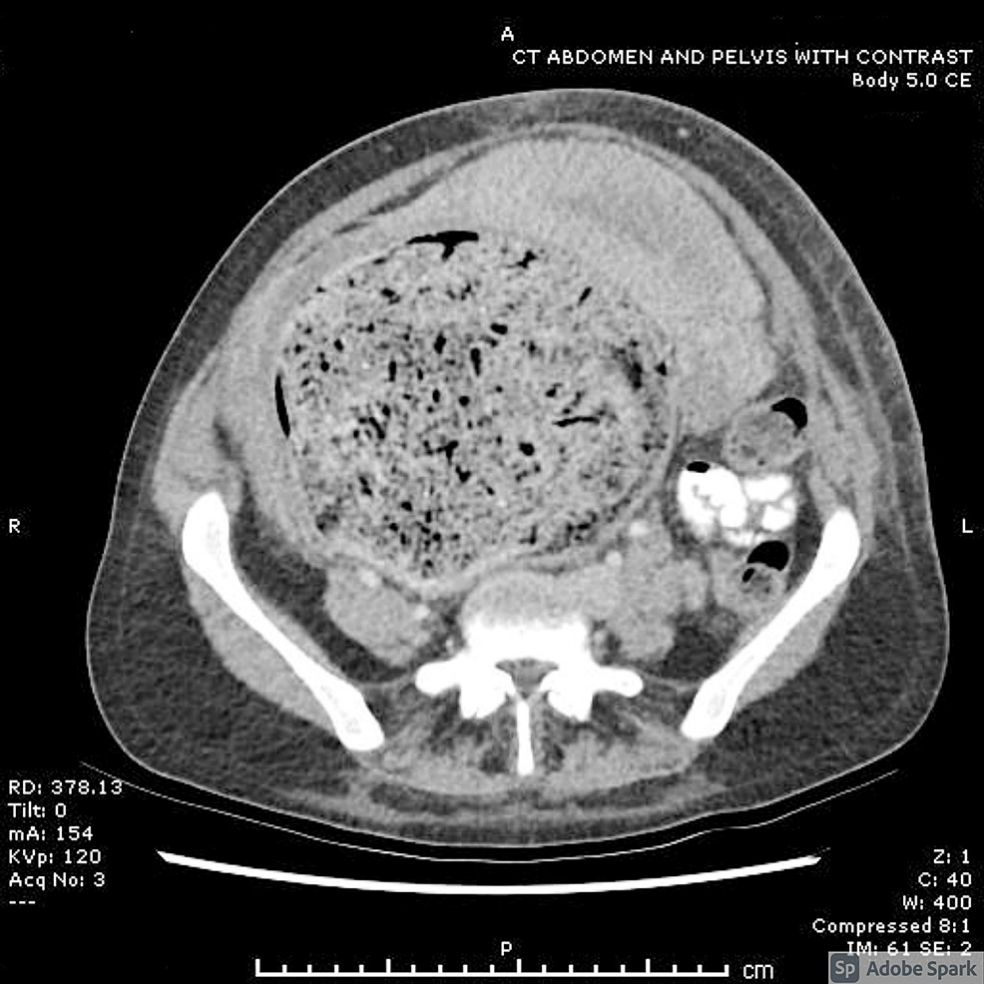
CT abdomen and pelvis - note uterus displaced by dilated bowel.

A 26-year-old pregnant woman, G4P2+1, was admitted with preterm premature rupture of membranes (PPROM) at 27 weeks. She was known to have chronic constipation since childhood leading to megarectum and megacolon (Figure 1). This required manual bowel evacuation and proctowash on multiple occasions. She had had two cesarean deliveries, first in 2015 for obstructed labor, followed by a second elective cesarean in 2019 during which there was grossly distorted anatomy secondary to abdominopelvic mass due to megarectum and megacolon requiring a T incision on uterus for delivery of baby, complicated by injury to the vagina. She was advised against future pregnancies.

During her current admission in 2019, she was 27 weeks pregnant and presented with PPROM, and the speculum examination was limited by impacted feces in megarectum completely obstructing the vagina. Laxatives and enemas failed to relieve constipation, thus requiring manual evacuation of bowel and proctowash with warm saline under spinal anesthesia. On the first postoperative day following the above procedure, within 6 hours patient got acutely unwell with chest tightness, cough, and rapidly worsening shortness of breath. She was tachypneic with low saturations, crepitations, brisk reflexes, and palpable liver edge.

Investigations revealed anemia with a low hematocrit, low fibrinogen, normal renal and liver functions, and inflammatory markers. An echocardiogram showed 55% ejection fraction and CT pulmonary angiogram (CTPA) showed that there was pulmonary edema with no evidence of embolism. A diagnosis of pulmonary edema was made; however, the etiology was unclear. It was queried to be due to anemia, fluid overload, sepsis secondary to PPROM, or atypical presentation of pre-eclampsia. In pregnant women, pulmonary edema occurs most commonly as a complication of severe pre-eclampsia. The classic triad of pre-eclampsia is hypertension, proteinuria, and edema. Recent data suggest that in some women, pre-eclampsia and even eclampsia may develop in the absence of hypertension or proteinuria, referred to as "atypical pre-eclampsia-eclampsia". Our initial suspicion was atypical pre-eclampsia presenting with pulmonary edema.

She was treated supportively with diuretics. However, due to the increasing requirement for oxygen and respiratory support, the decision was taken to deliver for maternal indication. The patient had a classical cesarean under general anesthesia with no intraoperative complications. Postoperatively she required intensive treatment unit care for one day and had a good postoperative recovery.

## Discussion

Our patient presented with PPROM at 27 weeks and was being managed conservatively. She required bowel evacuation and washout due to chronic constipation. There was sudden deterioration on the night of her bowel evacuation with acute onset of shortness of breath and tachypnea. Chest X-ray showed development of pulmonary edema and a fall in her hemoglobin to 69 g/dl from 93 g/dl preoperatively. We investigated her keeping in mind the differential diagnosis of pre-eclampsia, pulmonary embolism, sepsis, and cardiac condition as the cause for pulmonary edema. Our initial suspicion was pulmonary edema as a presentation of atypical pre-eclampsia. Multidisciplinary input was sought and on liaison with acute medicine and anesthetic colleagues, the above were excluded after normal pre-eclampsia blood and sepsis markers, a normal echocardiogram, and CTPA showing normal pulmonary vessels. In addition, there was a drop in hematocrit, which pointed out to hemodilution. Fluid overload from proctowash was thought to be the cause of pulmonary edema.

Pulmonary edema can be broadly classified as cardiogenic or non-cardiogenic. Cardiac output increases very early on and is the highest in the postpartum period. Plasma volume expands due to sodium and water retention, thereby increasing preload, but afterload reduces due to vasodilation [3]. Pregnancy is accompanied by physiological adaptations, making pregnant women more prone to developing pulmonary edema. Pulmonary vascular resistance, like systemic vascular resistance, decreases significantly in normal pregnancy. The colloid osmotic pressure/pulmonary capillary wedge pressure gradient is reduced by about 30%, making pregnant women particularly susceptible to pulmonary edema. Pulmonary edema will be precipitated if there is either an increase in cardiac preload (such as infusion of fluids) or increased pulmonary capillary permeability (such as in pre-eclampsia) or both [4].

In our case, a large amount of fluid used for irrigation, on the background of physiological changes of pregnancy, led to intravasation and third spacing of fluid. Fluid totals were not calculated during surgery. Most cases of acute pulmonary edema in pregnancy have been in association with pre-eclampsia, sepsis, or cardiac disease. Iatrogenic pulmonary edema due to surgery during pregnancy is rare. There is a reported case of pulmonary edema following fetoscopic umbilical cord coagulation for TRAP (twin reversed arterial perfusion) sequence due to fluid overload [5].

## Conclusions

Hemodynamic alteration in pregnancy makes pregnant women more susceptible to pulmonary edema while undergoing non-obstetric procedures. Our case illustrates complication of pulmonary edema after a low-risk procedure of manual bowel evacuation with proctowash. Unknown to us during the surgery, our patient had absorbed the normal saline, the irrigant, through the bowel. Our experience with this patient highlights the importance of the need for close liaison between obstetricians and other specialties in the management of pregnant women and astute monitoring and meticulous record of fluids during bowel washout.
